# Readmissions After Surgery for Colorectal Liver Metastases: A Propensity Score Analysis From the Colorectal Liver Operative Metastasis International Collaborative (COLOMIC)

**DOI:** 10.1002/wjs.70411

**Published:** 2026-06-12

**Authors:** Heidy Cos, Cristian D. Valenzuela, Omeed Moaven, Grey Leonard, John Stauffer, Nico R. Del Piccolo, Tan To Cheung, Carlos U. Corvera, Andrew Wisneski, Charles Cha, Kathleen Cummins, Greg Russell, Perry Shen

**Affiliations:** ^1^ Atrium Health Wake Forest Baptist Medical Center Winston Salem North Carolina USA; ^2^ Mayo Clinic in Florida Jacksonville Florida USA; ^3^ University of Hong Kong Pok Fu Lam Hong Kong; ^4^ University of California San Francisco San Francisco California USA; ^5^ Yale School of Medicine New Haven Connecticut USA

**Keywords:** colon cancer, liver metastases, readmissions

## Abstract

**Background:**

Readmission is considered as a surgical quality indicator. More data regarding predictors of readmission and outcomes after surgery for colorectal liver metastasis are needed.

Using a propensity score match to create a 1:2 match of cases:controls for 90‐day.

Readmission, the matching variables used for balance included age, tumor size, estimated blood loss, and type of resection (minor or major). T‐tests were used for continuous measures, Fisher's exact test was used for categorical data, and the Kaplan–Meier method was used to estimate survival. *p* < 0.05 was considered significant.

**Methods:**

Retrospectively examined 935 patients with CLM from 2000 to 2018.

Using a propensity score match to create a 1:2 match of cases:controls for 90‐day.

Readmission, the matching variables used for balance included age, tumor size, estimated blood loss, and type of resection (minor or major). T‐tests were used for continuous measures, Fisher's exact test was used for categorical data, and the Kaplan–Meier method was used to estimate survival. *p* < 0.05 was considered significant.

**Results:**

935 patients were included in the initial sample with 896 eligible for inclusion in the propensity matching. The average age of the population was 59% and 59% male. Overall readmission rate was 8.0%. Median time to readmission was 14 days. In the propensity score matched sample, readmitted patients had higher rates of organ space infection (28% vs 2% and *p* < 0.0001), bile leak (26% vs 1% and *p* < 0.0001), and liver failure (9% vs 2% and *p* = 0.042). There was no difference in LOS (7 days versus six days and *p* = 0.40), overall survival (median 39.7 vs 41.0 months and *p* = 0.79), or rate of adjuvant therapy (68% for both and *p* > 0.99).

**Conclusion:**

Patients readmitted for intermediate and late complications after surgery for CLM can recover and receive adjuvant therapy with no adverse effect on overall survival. Organ space infection, bile leak, and liver failure are highly associated with readmission.

## Graphical abstract

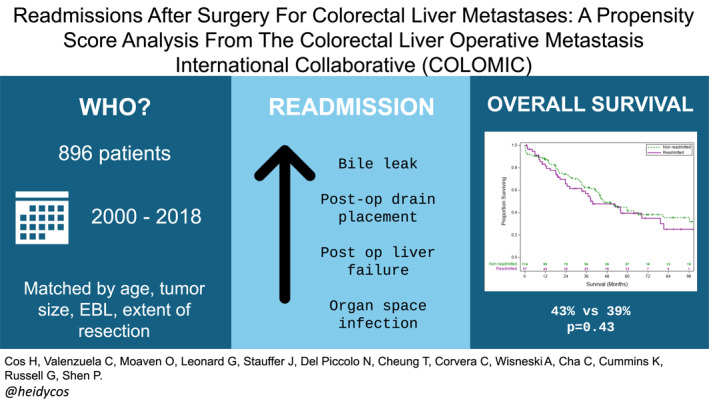

## 1 Introduction

Resection of colorectal liver metastases (CLM) has been shown to improve survival in this patient population [1, 2, 3, 4, 5]. With improved survival and higher operative volumes, hospital readmission rates have become an important outcome, as well as a surgical quality metric in this population [6, 7, 8]. Although readmissions are often thought of as an adverse event, readmissions allow for documentation of mid‐to‐late postoperative complications and offer a window of opportunity to rescue patients from worse morbidity or mortality [9].

Readmission after hepatectomy for any diagnosis is approximately 10.5% [10, 11], and ranges 13%–46% after liver surgery for CLM [12, 13, 14]. However, there is limited data available on specific factors related to readmissions after hepatectomy for CLM. Our Colorectal Liver Operative Metastasis International Collaborative (COLOMIC), a longitudinal multi‐institutional database with over one thousand patients spanning 18 years, is uniquely poised to define the risk factors for readmission in this population.

A more comprehensive approach to measure surgical quality is by evaluating textbook outcomes [15]. Textbook outcome for a particular surgery involves a composite score based on the ideal postoperative experience. In liver surgery, it has been defined as the absence of intraoperative incidents of grade 2 or higher (defined according to the Oslo classification), postoperative bile leak of grade B or C (according to the severity grading of the International Study Group of Liver Surgery), severe complications (Clavien–Dindo grade III or higher), postoperative reintervention (i.e., surgical, endoscopic, or radiologic), readmission within 30 days after discharge, in‐hospital mortality, and the presence of R0 resection margin (i.e., 1 mm or more tumor‐free margin) [16]. Others have proposed slight variations of textbook outcome in liver surgery to include the absence of a prolonged length of stay as well [17].

Though postoperative readmission is considered a component of surgical quality, other studies have reported on the importance of failure to rescue as the cause for mortality after major complex surgery [9]. Readmission for treatment of postoperative complications may be important for treating complications which are inherent in major abdominal surgery. Herein, we present a propensity score analysis looking at factors associated with and outcomes of readmissions after surgery from colorectal liver metastases from the COLOMIC database.

## 2 Methods

The COLOMIC is a multi‐institutional database of patients with CLM treated with hepatectomy from the following institutions: Atrium Health Wake Forest Baptist Medical Center (AH‐WFBMC), Mayo Clinic Florida, University of California San Francisco (UCSF), Yale New Haven Hospital, and the University of Hong Kong. It includes consecutive cases from 2000 to 2018 (*n* = 1004) at all participating institutions. IRB approval was obtained from all participating institutions for this study. Patients must have received a curative‐intent hepatectomy operation (major and minor and anatomic and nonanatomic resections, including those that may have incorporated hepatic ablations). Major resection was any standard right or left hepatectomy as well as extended right or left hepatectomy, in addition to any resection of 3 segments or greater. Patients who received ablation‐only procedures without hepatectomy were excluded. All technical methods of liver resection (crush‐clamp, energy device, or hybrid) were included. Liver failure was defined using the International Study Group of Liver Surgery (ISGLS) guideline for posthepatectomy liver failure [18]. Bile leak was defined using the International Study Group of Liver Surgery (ISGLS) guideline for posthepatectomy bile leak as follows: fluid with an elevated bilirubin level in the abdominal drain or intra‐abdominal fluid on or after postoperative day three or the need for radiological intervention (i.e., interventional drainage) owing to biliary collections or re‐laparotomy due to biliary peritonitis. The elevated bilirubin level in the drain or intra‐abdominal fluid is defined as a bilirubin concentration at least three times higher than the serum bilirubin level measured at the same time [19].

### 2.1 Statistical Methods

We performed a retrospective review of 935 patients with colorectal liver metastases who underwent surgery between 2000 and 2018 and evaluated their 90‐day readmission rates. Of these 935, 39 were removed prior to matching due to incomplete data on survival status postsurgery. Summary statistics, including frequencies and proportions for categorical outcomes and means and standard deviations for continuous variables, were calculated for all study measures. For our primary analysis, propensity score matching was performed in a 1:2 ratio of cases to controls (readmitted to nonreadmitted, respectively), controlling for the four variables that were significantly associated with the likelihood of readmission: patient age, tumor size, estimated blood loss, and resection status (major or minor). Other candidate variables that were not associated with readmission included Charlson–Deyo Comorbidity Index, intraoperative drain placement, neoadjuvant chemotherapy, and surgical approach [20]. This resulted in a matched dataset of 57 cases and 114 controls. To assess the propensity matching results, the standardized mean difference (SMD) was calculated for the four selected matching variables [21]. The SMD was < 0.1 for each variable indicating an acceptable balance between groups. For assessing differences between cases and controls, Fisher's exact test was used for categorical variables and Student's t‐test was used for continuous variables. The Kaplan–Meier method, using the log‐rank test of the chi‐squared approximation to assess statistical significance, was used for estimating overall survival. *p*‐values < 0.05 were considered statistically significant. SAS (version 9.4, Cary, NC, USA) was used for all analyses.

## 3 Results

A total of 896 patients underwent hepatectomy for colorectal liver metastases in the study period. Fifty‐nine percent were males with a mean age of 59. The mean Charlson–Deyo comorbidity score was 8.1, with all patients receiving a baseline of 6 given the presence of metastatic disease (SD 1.4). Eighty‐seven (9.7%) patients had extrahepatic disease on PET, 250 (27.9%) patients had bilobar disease, the mean number of lesions was 2.3 (SD 2.1) with a mean size of 37.3 mm (SD 29.1), and 2.8% of patients had cirrhosis on final pathology from the time of surgery. Of the 896, 357(39.9%) of hepatectomies included a major resection and 610 (68.2%) were performed via an open approach (Table 1).

**TABLE 1:** Characteristics of the overall population (*N* = 896).

Characteristic	Result
Age (mean, SD)	59.9 (11.5)
Male sex (N, %)	526 (58.7)
Race (N, %)	
Caucasian	553 (61.7)
African American	80 (8.9)
Asian	218 (24.3)
Other	45 (5.0)
BMI (mean, SD)	26.8 (5.8)
ASA	
1	38 (4.3)
2	303 (34.3)
3	513 (58.0)
4	31 (3.5)
Charlson score (mean, SD)	8.1 (1.4)
Extrahepatic disease on PET (N, %)	87 (9.7)
Bilobar disease (N, %)	250 (27.9)
Number of lesions (mean, SD)	2.3 (2.1)
Cirrhosis (N, %)	25 (2.8)
Major resection (N, %)	357 (39.9)
Open approach (N, %)	610 (68.2)
Steatosis (N, %)	196 (21.9)
Tumor size, mm (mean, SD)	37.3 (29.1)
Neoadjuvant chemotherapy (N, %)	480 (53.6)

There were 337 (37.6%) of patients who experienced at least one complication. Of these, 4.5% were due to organ space infections. There were 28 bile leaks (3.1%), 10 patients developed liver failure (1.1%), and 31 patients required reoperation (3.5%). The average length of stay (LOS) was 7.3 days (SD = 8.7) and the readmission rate was 7.9% (Table 2). The median overall survival for the population was 44.3 months, and 66.6% of patients received adjuvant therapy.

**TABLE 2:** Outcomes of the overall population (*N* = 896).

Outcomes	Result
Complications (*N*, %)	337 (37.6)
Organ space infection (*N*, %)	40 (4.5)
Bleeding (*N*, %)	15 (1.7)
Bile leak (*N*, %)	28 (3.1)
Liver failure (*N*, %)	10 (1.1)
Reoperation (*N*, %)	31 (3.5)
LOS (mean, SD); median [p25, p75]	7.3 (8.7); 6 [4, 8]
Readmission (*N*, %)	71 (7.9)
Survival, months (median)	44.3
Adjuvant therapy (*N*, %)	597 (66.6)

For the propensity score analysis, the control group (no readmission) included 114 patients and the readmission group included 57 patients. Although there were 71 readmissions due to missingness on estimated blood loss (*n* = 11) and/or tumor size (*n* = 6), 14 readmitted patients were not able to be matched. Patients were matched without any adjustment for mortality; there were 0 deaths in the readmission group and 8/114 in the no readmissions group who died within 30 days. Six of these deaths occurred in‐hospital during index admission. The rate of mortality is not significantly different between groups (*p* = 0.053). There were no significant differences in demographic characteristics between the two groups (Table 3). In the control group, 29 (25.4%) were treated at Mayo Clinic Florida, 38 (33.3%) were treated in Hong Kong, 23 (20.2%) at AH‐WFBMC, 17 (14.9%) at UCSF, and 7 (6.1%) at Yale. In the readmitted group, 14 (24.6%) were treated at Mayo Clinic Florida, 10 (17.5%) were treated in Hong Kong, 16 (28.1%) at AH‐WFBMC, 13 (22.8%) at UCSF, and 4 (7.0%) at Yale. There was no difference in rates of KRAS mutation between the readmitted and not readmitted patients (14% vs 18% and *p* > 0.99). Most primary tumors were left sided (61% in not readmitted vs 65% in readmitted and *p* = 0.83) and in both groups, most patients had their primary tumor resected before their hepatectomy (76% in readmitted group, 81% no readmission group, and *p* = 0.53).

**TABLE 3:** Characteristics comparison readmitted versus not readmitted; propensity score matched populations (*N* = 171).

Characteristic	No readmit (*N* = 114)	Readmit (*N* = 57)	*p* value
Male sex (N, %)	71 (62)	35 (61)	> 0.99
Race (N, %)			0.015
Caucasian	65 (57)	33 (58)	
African American	6 (6)	11 (19)	
Asian	40 (35)	11 (19)	
Other	2 (2)	2 (4)	
BMI (mean, SD)	26.8 (5.6)	26.8 (5.4)	0.99
Smoking history (*N*, %)	34 (30)	18 (32)	0.86
Charlson score (mean, SD)	7.9 (1.5)	7.8 (1.4)	0.75
Extrahepatic disease on PET (*N*, %)	10 (9)	4 (7)	0.78
Portal vein embolization (*N*, %)	8 (7)	7 (12)	0.26
Bilobar disease (*N*, %)	37 (32)	13 (23)	0.22
Major resection (*N*, %)	63 (55)	28 (49)	0.63
Steatosis (*n*, %)	16 (14)	13 (23)	0.19
Cirrhosis (*N*,%)	5 (4)	3 (5)	> 0.99
Size of tumor (mean, SD)	47.0 (39.5)	44.4 (39.5)	0.69
EBL, ml [median (p25, p75)]	500 (200, 1000)	500 (150, 1150)	0.84
Neoadjuvant chemotherapy	59 (52)	35 (61)	0.26
KRAS mutant	26/60 (43)	9/24 (37)	0.81
MSI high	0/16 (0)	0/6 (0)	> 0.99
Colon tumor laterality			0.83
Right	43 (38)	20 (35)	
Left	70 (61)	37 (65)	
Bilateral	1 (1)		
Intraoperative drain placement	31/92 (34)	16/46 (35)	> 0.99

The superficial surgical site infection rate was 4% in the control group and 9% in the readmission group (*p* = 0.16). The rate of organ space infection was higher in the readmitted patients (28% vs 2% and *p* < 0.0001). There were 15 bile leaks in the readmitted group compared to 1 in the control group (26% vs 1% and *p* < 0.0001). Thirty‐two patients in the readmission group required a drain placement postoperatively compared to 7 patients in the no readmission group (56% vs 6% and *p* < 0.0001). See Table 4.

**TABLE 4:** Outcomes comparison readmitted versus not readmitted; propensity score matched populations (*N* = 171).

Outcome	No readmit (*N* = 114)	Readmit (*N* = 57)	*p* value
Superficial SSI (*N*, %)	4 (4)	5 (9)	0.16
Deep SSI (*N*, %)	1 (1)	2 (4)	0.26
Organ space infection (*N*, %)	2 (2)	16 (28)	< 0.0001
Bleeding (*N*, %)	0 (0)	3 (5)	0.036
Bile leak (*N*, %)	2 (2)	15 (26)	< 0.0001
Postop drain placement *(N*, %)	7 (6)	32 (56)	< 0.0001
Postop liver failure (*N*, %)	1 (1)	5 (9)	0.016
Reoperation (*N*, %)	1 (1)	10 (17)	< 0.0001
LOS (median (p25, p75))	6 (5, 9)	7 (5, 9)	0.66
Days to readmission (median (p25, p75))	NA	15 (9, 32)	

Five (9%) patients in the readmissions group versus one (1%) in the control group developed postoperative liver failure (*p* = 0.016). Seventeen percent of readmitted patients required reoperation compared to 1% of those who were not readmitted, *p* < 0.0001. The median LOS at index admission was similar in both groups: 7 versus 6 days (*p* = 0.66). For the readmission group, median time to readmission after discharge was 14 days (range 0–81). See Table 4.

Patients with larger tumors were more likely to develop a bile leak. Moreover, those who underwent a major liver resection were significantly more likely to develop postoperative liver failure. Higher number of lesions before surgery was also associated with postoperative liver failure. See Table 5.

**TABLE 5:** Perioperative characteristics of patients with organ space infection, bile leak, and postoperative liver failure; propensity score matched populations (*N* = 171).

Variable	Organ space infection		*p*‐value
	No (*n* = 153)	Yes (*n* = 18)	
Female sex	37.9%	38.9%	> 0.99
Left lobe lesions	20.3%	11.1%	0.074
Bilobar lesions	31.4%	11.1%	
Right lobe lesions	48.4%	77.8%	
Cirrhosis	3.9%	11.1%	0.20
Major resection	52.9%	61.1%	0.62
Open surgery	70.6%	77.8%	0.60
Neoadjuvant chemotherapy	52.3%	77.8%	0.047
Age, median (p25, p75)	59 (50, 68)	56 (46, 65)	0.32
BMI	25.8 (23.1, 29.8)	27.8 (24.1, 32.3)	0.42
ASA	3 (2, 3)	3 (3, 3)	0.28
Number of lesions	2 (1, 3)	2 (2, 5)	0.093
Size of tumor	35 (21, 60)	40 (17, 50)	0.83

**Variable**	**Bile leak**		***p*‐value**
	**No (*n* = 154)**	**Yes (*n* = 17)**	
Female sex	38.3%	35.3%	> 0.99
Left lobe lesions	20.1%	11.8%	0.32
Bilobar lesions	30.5%	17.6%	
Right lobe lesions	49.4%	70.6%	
Cirrhosis	5.2%	0.0%	> 0.99
Major resection	53.2%	58.8%	0.80
Open surgery	70.8%	76.5%	0.78
Neoadjuvant chemotherapy	54.5%	58.8%	0.80
Age, median (p25, p75)	59 (50, 67)	54 (50, 59)	0.17
BMI	25.9 (22.9, 29.8)	25.8 (24.6, 32.0)	0.33
ASA	3 (2, 3)	3 (3, 3)	0.53
Number of lesions	2 (1, 3)	2 (1, 4)	0.52
Size of tumor	31 (20, 54)	42 (37, 70)	0.036

**Variable**	**Postop liver failure**		***p*‐value**
	**No (*n* = 165)**	**Yes (*n* = 6)**	
Female sex	37.6%	50.0%	0.67
Left lobe lesions	20.0%	0.0%	0.33
Bilobar lesions	29.7%	16.7%	
Right lobe lesions	50.3%	83.3%	
Cirrhosis	4.8%	0.0%	> 0.99
Major resection	52.1%	100.0%	0.031
Open surgery	70.3%	100.0%	0.18
Neoadjuvant chemotherapy	54.5%	66.7%	0.69
Age, median (p25, p75)	58 (50, 67)	58.5 (58, 59)	0.93
BMI	25.8 (23.1, 29.9)	29.7 (26.6, 32.0)	0.30
ASA	3 (2, 3)	3 (3, 3)	0.13
Number of lesions	2 (1, 3)	4.5 (2, 11)	0.036
Size of tumor	35 (20, 55)	55 (30, 78)	0.18

There was no statistically significant correlation between readmission and grade of complications (Table 6).

**TABLE 6:** Correlation between complication grade (by Clavien–Dindo) and readmission.

Complication Grade	Readmission			
	No (*N* = 114)	Yes (*N* = 57)	Total	*p*‐value
0	86 (75%)	36 (63%)	122	0.10
1	16 (14%)	15 (26%)	31	
2	9 (8%)	5 (9%)	14	
3	0 (0%)	1 (2%)	1	
4	3 (3%)	0 (0%)	3	

**FIGURE 1:**
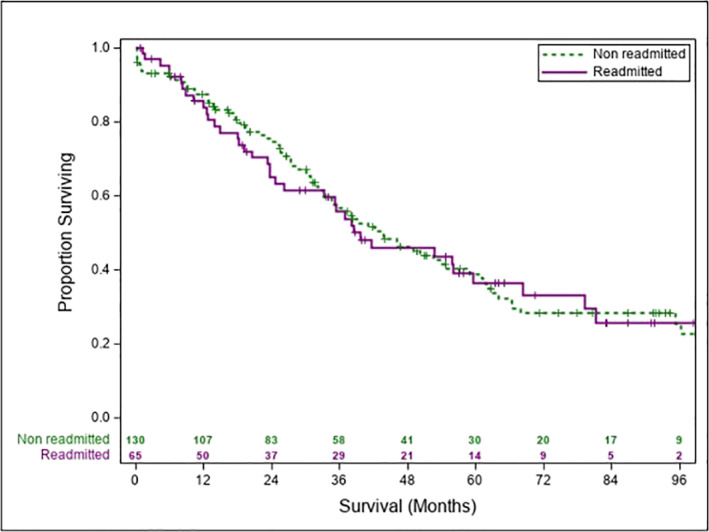
The median survival in the readmission group was 39.7 months compared to 46.6 months in the control group, *p* = 0.43.

The median survival in the readmission group was 39.7 months compared to 46.6 months in the control group, *p* = 0.43 (Figure 1). The rate of adjuvant therapy was also similar in both groups (67% vs 68% and *p* = 0.86). The 1‐, 3‐ and 5‐year survival rates for both groups are presented in Table 7.

**TABLE 7:** Survival outcomes; propensity score matched population (*N* = 171).

Survival	No readmit (*N* = 114)	Readmit (*N* = 57)	*p* value
1 year	87.6% (SE 3.2)	81.5% (SE 5.3)	0.43
3 years	62.4% (SE 5.2)	57.0% (SE 7.0)	
5 years	43.1% (SE 5.3)	39.5% (SE 7.4)	
Median	3.9 years (46.6 months)	3.3 years (39.7 months)	
Adjuvant therapy	76 (67)	39 (68)	0.86

## 4 Discussion

Several retrospective reviews exist on outcomes after liver surgery for CLM; however, there is little reported data on readmissions after hepatectomy in this context. Our study shows a readmission rate of 7.9% after hepatectomy for CLM, which is lower than other similar published studies. Tamandl et al. [12] reported 13% readmission rate after liver surgery for CLM in 746 patients. However, almost 20% of their patients had ablation‐only procedures, which may have skewed the results since these patients are more likely to have outpatient procedures.

Jin and colleagues [22] performed a propensity score matched comparison of outcomes after surgery for CLM in patients < 70 years old versus > 70 years old. In their study of 724 patients, they reported a readmission rate of 0.5% in the younger group and significantly higher, 4.1%, in the older group. Although we did not stratify our population by age, our readmission rate was slightly higher than that of the older population of Jin et al. Our study also had a higher proportion of major hepatectomy (39.9%) compared to 18% in their study, which was significantly associated with readmission.

The most common complications associated with readmission in our study were organ space infection, bleeding, bile leak, and postoperative liver failure. Organ space infection was associated with readmission in 28% of the patients in the propensity score matched analysis comparable to other reports [12, 14]. The bile leak rate in our overall population was 3.1% and was present in 26% of readmitted patients. Egeland and colleagues [14] reported a 4% bile leak rate; however, patients were not stratified by the readmission status. In their distribution of complications between primary stay and readmission, Tamandl et al. [12] reported 18% of bile leaks were treated during index admission and 10% at readmission.

These studies did not comment on surgeons' practices regarding surgical drain as it might be an indication of a surgeon's bias toward a higher risk case. It is well reported that drain do not prevent need for drainage procedures post operatively. A retrospective study by Squires and colleagues [23] examined 1041 patients who underwent major hepatectomies at 3 institutions from 2000 to 2012. In this study, drain placement after major hepatectomy did not decrease need for secondary drainage procedures. Similarly, Shahin and colleagues published a meta‐analysis from seven randomized controlled trials reporting 1064 patients undergoing hepatectomy with or without drain placement, concluding significantly higher overall complication rate and no difference in bile leaks [24]. Our study evaluated the 90‐day postoperative period for readmissions, whereas most existing studies only covered 30 days of postoperative follow‐up [12, 23, 25].

Ten patients (1.1%) in the overall population developed liver failure postoperatively, and this was observed in 9% of readmitted patients; however, our data were limited on the severity of liver failure in these cases. Approximately 2.8% of patients in the overall population had preexisting cirrhosis at the time of surgery as evidenced by final pathology reports from hepatectomy. In the readmitted group, 5% of patients had cirrhosis at the time of surgery compared to 4% of the nonreadmitted patients. In a pathologic study of over 400 livers of patients who underwent liver resection for CLM, Vauthey and colleagues found that steatohepatitis was associated with increased 90‐day mortality after liver surgery [26]. One could consider preoperative exposure to chemotherapy as a potential contributor to this outcome; however, the data available regarding preoperative chemotherapy in this patient population are limited to the rate of neoadjuvant chemotherapy by group (52% of nonreadmitted and 61% of readmitted) and lacks more granular data such as doses and extent of length of therapy.

Our study showed that there was no difference in return to other intended oncologic therapy regardless of readmission status. This database is limited by the absence of timing information, and thus, we cannot establish if there was an associated delay.

Our study has limitations due to its retrospective nature as well as clinical practice variations between the participating institutions. Although some readmissions could potentially have been avoided and complications managed in the outpatient setting, practices surrounding this type of management might differ and were not protocolized for the purposes of this study. Moreover, our database did not capture granular details of each readmission and management of complications. Additionally, specific details on neoadjuvant and adjuvant treatments (such as timing) were not included, which could have been helpful in describing associations of these treatments to readmissions.

In conclusion, approximately 7.9% of patients who undergo resection of CLM with hepatectomy required readmission within 90 days of surgery. Organ space infection, bile leak, and liver failure were associated with higher readmission rates. Readmission may be an indicator of ‘rescue’ from late complications. Patients who were readmitted had similar long‐term overall survival compared with patients who were not readmitted. This study showed that patients with larger tumors, higher number of liver lesions, and those undergoing major hepatectomy were at increased risk for severe complications. These patients may benefit from earlier postoperative follow‐up and/or structured postoperative surveillance protocols including planned home health visits with increased frequency and regular check‐in calls.

## Author Contributions

**Tan To Cheung:** investigation, funding acquisition, supervision. **Nico Del Piccolo R:** investigation, funding acquisition, methodology, supervision. **John Stauffer:** investigation, methodology, funding acquisition, supervision. **Charles Cha:** investigation, funding acquisition, supervision. **Cristian D: Valenzuela:** validation, supervision, methodology. **Grey Leonard:** investigation, methodology, supervision. **Kathleen Cummins:** investigation. **Omeed Moaven:** investigation, methodology, supervision. **Heidy Cos:** conceptualization, investigation, writing–riginal draft, methodology, writing–review and editing. **Greg Russell:** methodology, investigation, formal analysis, data curation, writing–original draft, writing–review and editing. **Andrew Wisneski:** investigation, funding acquisition, supervision. **Carlos Corvera U:** investigation, funding acquisition, supervision. **Perry Shen:** conceptualization, investigation, funding acquisition, writing–original draft, writing–review and editing, supervision.

## Funding

This work was supported in part by Wake Forest University Comprehensive Cancer Center. Our Biostatistics support was a shared resource funded via the NCI grant award P30CA012197.

## Conflicts of Interest

The authors declare no conflicts of interest.

## Data Availability

The data that support the findings of this study are available from the corresponding author upon reasonable request.
